# A Schema-Based Robot Controller Complying With the Constraints of Biological Systems

**DOI:** 10.3389/fnbot.2022.836767

**Published:** 2022-05-09

**Authors:** Fabien Lagriffoul

**Affiliations:** Centre for Applied Autonomous Sensor Systems (AASS), Orebro University, Örebro, Sweden

**Keywords:** sensorimotor schema, predictive model, learning, developmental robotics, Piaget, constructivism

## Abstract

This article reports on the early stages of conception of a robotic control system based on Piaget's schemas theory. Beyond some initial experimental results, we question the scientific method used in developmental robotics (DevRob) and argue that it is premature to abstract away the functional architecture of the brain when so little is known about its mechanisms. Instead, we advocate for applying a method similar to the method used in model-based cognitive science, which consists in selecting plausible models using computational and physiological constraints. Previous study on schema-based robotics is analyzed through the critical lens of the proposed method, and a minimal system designed using this method is presented.

## 1. Introduction

Developmental robotics (DevRob) is the logical outcome of convergent ideas from different fields in Cognitive Science. In Philosophy, the theory of Embodied Cognition (EC) (Varela et al., 1992) proposes an alternative to the Classical Computational Theory of Mind (CTM) (Fodor, 1975; Pylyshyn, 1984), rejecting the view of intelligence as a symbol manipulation process where the symbols are abstract representations detached from the organism and its environment. Cognition does not take place only in the brain but through the continuous process of interaction between mind, body, and environment. In Robotics, the *Nouvelle AI* movement pioneered by Brooks (1990) challenges classical symbolic AI, questioning the capacity of abstract symbols to represent and reason upon the world. Instead, it is argued that intelligence emerges from the interaction of multiple components implementing simple sensorimotor couplings between an agent and the environment. In Psychology, Piaget introduces a constructivist theory of knowing, in which learning takes place stage-wise, through the interaction of the child with her environment (Piaget and Cook, 1952). Unlike the behaviorist approach which focuses and different types of conditioning, Piaget emphasizes the active construction of novel structures to make sense of new experiences. DevRob (Guerin, 2011; Cangelosi and Schlesinger, 2014) aims at designing robotic systems along with these principles, which could be summarized by two key ideas: (i) knowledge is *constructed* through the interaction of the robot and its environment, and (ii) the focus of interest is not to “reverse-engineer” intelligence but rather to understand the principles that allow it to *develop*.

However, despite an appealing theoretical framework, DevRob has not made significant progress when compared to “mainstream” approaches to Robotics, which have leveraged technical advances in other AI fields like Deep Learning. For the remainder of the discussion, the theoretical framework of cognitive development by Piaget (Piaget and Cook, 1952; Piaget, 1971) will be assumed. According to Asada et al. (2009), DevRob suffers from a double problem: higher order cognitive functions depend on lower primary functions, therefore, building a developmental system requires a good understanding of both primary functions *and* developmental mechanisms. Let us examine each element separately. In Piaget's framework, the primary functions are the *sensorimotor schemas*. Although sensorimotor schemas are not formally defined in the theory, there is abundant neuroscientific literature about sensorimotor phenomena upon which roboticists can draw. The case is different for developmental mechanisms, for which little knowledge is available since most studies address steady state functions of the brain, rather than how they develop through time (Karmiloff-Smith, 1996). On the theoretical side, Piaget's theory provides overarching principles, and three abstract mechanisms (assimilation, accommodation, and equilibration), which are too vague about how to concretely implement a developmental mechanism. This lack of knowledge is reflected in previous study on “Piagetian” robotics: there is an overall agreement on how to model sensorimotor schemas (typically, a learned action model), but the picture is less clear for developmental mechanisms. These are neither grounded on theoretical nor empirical models, which often results in implementing some *ad hoc* mechanisms designed to fit with the other components of the system. Admittedly, DevRob is an experimental field; “trying out” and comparing different ideas might lead to interesting insights, but the lack of guiding principles might also lead to a dispersion of efforts. This raises the question of the scientific method to use in DevRob, which we discuss next.

**Figure 1:**
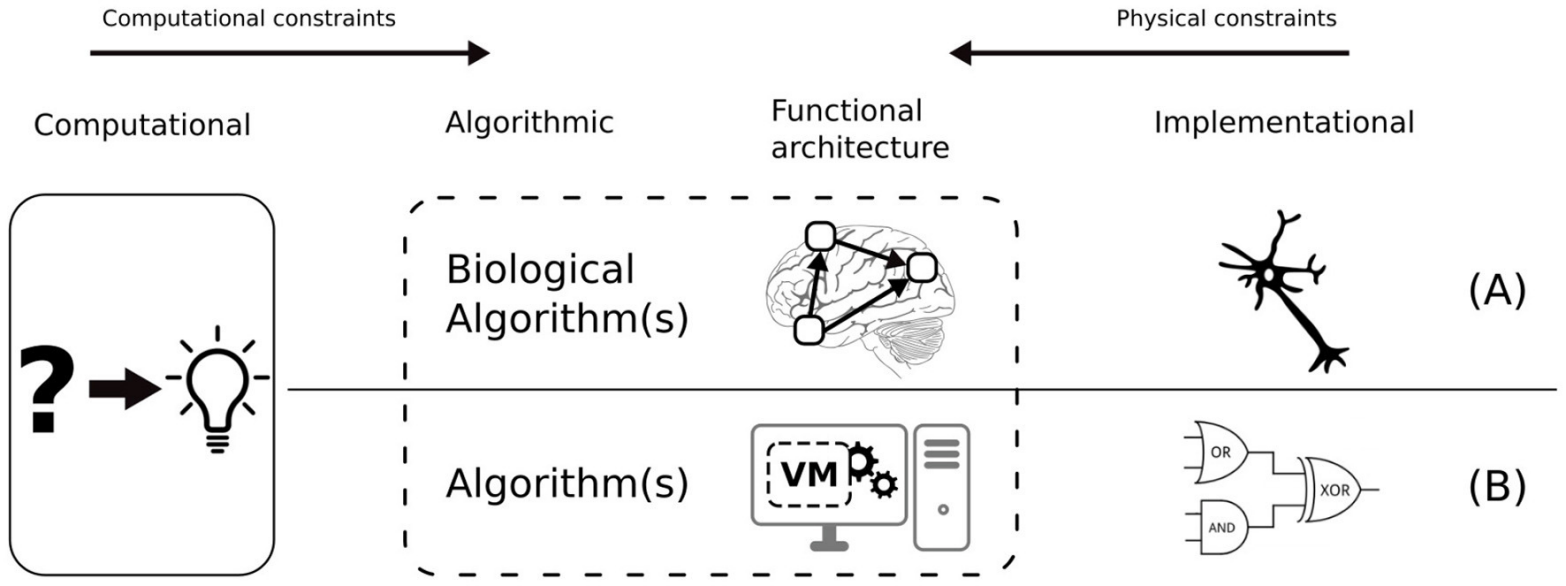
Marr's three levels of analysis: Computational, Algorithmic, and Implementational from a biological **(A)** and artificial **(B)** perspective. Also, the functional architecture: the basic functions of the functional architecture cannot be decomposed further without considering implementational details.

The “grand challenge” of cognitive science is to bridge the different levels of analysis of the human mind, i.e., computational, algorithmic, and implementational levels (Marr, 1982). Model-based cognitive science creates mathematical models of cognition and tests them by comparing the predictions of these models to empirical data (typically response times and accuracy) (Griffiths et al., 2010). From the bottom up, cognitive neuroscience uses neural imaging data to constrain computational models (Palmeri et al., 2017). The range of plausible explanations is narrowed down by the *constraints* imposed across the different levels of analysis (refer to Figure 1A). It is important to note that the algorithmic level is tightly connected to *functional architecture*, i.e., a set of primitive operations, representations and processes, upon which algorithms can be specified (Pylyshyn, 1984). By analogy with computers, functional architecture is a kind of virtual machine on which “cognitive algorithms” are executed. At the implementational level, neural wetware and computer hardware are so different that one must consider this interface to some extent when designing a biologically-inspired system (refer to Figure 1B).

A good analogy for illustrating the importance of functional architecture is Artificial Neural Networks (ANN) (McCulloch and Pitts, 1943). The mode of operation of ANNs intrinsically depends on their functional architecture. Even though ANNs are implemented on computers using serial processing, the functional architecture of biological neural networks is preserved in several aspects: (i) high-dimensional raw input, (ii) distributed processing of the input through hidden neurons, (iii) parallelism at the layer level, i.e., all the outputs of a layer must be computed before feeding the next layer. Obviously, there exist mathematical methods that can achieve a similar *function* (e.g., classifying input data) using algorithms that are intrinsically serial, e.g., Support-vector networks (Cortes and Vapnik, 1995). The point is that functional modeling is a sensible approach as far as the intended function—in this case, classifying data—is clearly defined. But in the case of sensorimotor schemas (or cognitive systems in general), the functionality is not well defined nor understood. Our stance in such a situation is that the functional architecture should be preserved as much as possible, to avoid overlooking computational properties which are not yet understood.

Currently, our knowledge of the functional architecture of the brain is not complete, which results in a chicken-egg situation for specifying algorithms, since basic operations depend on the functional architecture. In our opinion, our current understanding of brain function is too limited to do functional modeling independently of a plausible supporting architecture. We argue that it results in an ineffective trial-and-error design cycle and that it is more effective to account for biological constraints until more is known about brain function. Therefore, one should aim at systems *strongly equivalent* to their biological counterparts, i.e., systems which run similar algorithms, using similar representations and processes, on a similar functional architecture. For this reason, we shall use a method inspired by the “constraint-based” method used in model-based cognitive science, which consists in constraining the algorithmic level with biologically plausible architectural constraints.

More specifically, we shall consider two types of constraints inherent to biological systems: constraints on data representation and constraints on processing. These constraints are rarely accounted for in functional modeling approaches, which use algorithms developed for computer architectures. Biological systems use distributed representations and process data in a parallel or concurrent manner, whereas classical computer methods use compact representations (vectors, symbols) and serial processing (conditional statements, loops). Our proposed approach is to build a minimal cognitive agent based on a simple schema mechanism, which complies with the aforementioned biological constraints, i.e., distributed representations and non-serial processing.

The proposed method is not novel and in some ways, it is self-evident. Nevertheless, from the succinct literature review presented in Section 2, we argue that this method can operate a drastic selection among candidate algorithms, by showing in Section 3 that all the reviewed studies do not comply with some basic constraints derived from well-established neuroscientific facts. In Section 4, we introduce several versions of a minimal cognitive agent using such architectural constraints, which will serve as a developmental “seed” for future study. From the preliminary results presented in Section 5, we discuss some possible directions for future study in Section 7.

## 2. Previous Study

The monumental study by Piaget is out of the scope of the article, but introducing some key concepts might give some context before introducing the related study in robotics. Piaget is well-known for his work on developmental stages and the construction of knowledge in children (Piaget and Cook, 1952), but his constructivist prism was also applied to a wide range of phenomena, including biological development (Piaget, 1971) and scientific progress (Piaget et al., 1988). The theoretical starting point for roboticists is Piaget's schema theory. *Schemas* are the basic building blocks of knowledge. They represent different types of knowledge depending on the stage of cognitive development of the child, e.g., sensorimotor schemas during the sensorimotor stage, and more abstract schemas in the latter stages. Throughout development, infants acquire new schemas through the mechanisms of assimilation, accommodation, and equilibration. The process of assimilation takes new information into existing structures, while accommodation alters existing structures when assimilation is not possible. As assimilation and accommodation occur, the system gets unbalanced due to conflicts or internal contradictions, which are resolved by the process of equilibration.

Piaget's theory is not the unique entry point to DevRob; a wider perspective on DevRob can be found in the surveys by Lungarella et al. (2003) and Guerin (2011), and a more specific overview of previous study inspired by Piaget was done by Stojanov (2009). In the scope of this article, we focus on studies implementing Piaget's schema theory, and more specifically on those involving embodied agents.

One of the first systems inspired by Piaget's schema theory was proposed by Drescher (1987). Drescher's schemas represent a (*context, action*) → *result* relation, i.e., they learn a causal model of the actions of the agent in the world, unlike Reinforcement Learning which learns the *value* of the actions with respect to a state for a given task. The context is represented by a set of binary elements called *items* representing perceptual features. The system implements an inductive mechanism called *marginal attribution*, which identifies the items causing reliable state transitions (since actions may have unpredictable effects). Abstraction occurs by creating *synthetic items*, which augment the context with new (non-perceptual) concepts, and through *composite actions* which represent chains of primitive actions. The test bed for the schema mechanism is the “micro-world,” a grid-like environment in which the agent can interact with objects by controlling a symbolic hand and moving its visual field over the grid.

The CLASM system (Chaput et al., 2003; Chaput, 2004) resembles Drescher's schema mechanism, but the costly *marginal attribution* mechanism is replaced by hierarchical unsupervised learning with Self Organizing Maps (Kohonen, 2001). With this more efficient implementation, the system can deal with more complex environments, and it is applied to a foraging scenario with a robotic platform. The robot (real and simulated) is a mobile robot with a differential drive, a camera, and a gripper, which senses the environment through 5 “blob detectors” located in front of the robot. Drescher's schema mechanism has also inspired the CALM system (Perotto et al., 2007), which operates in a purely abstract domain.

Georgeon and Ritter (2012) proposed a schema-based developmental system exploiting the idea of intrinsic motivation. In this paradigm, the perception of the state and the value assigned to action are not objective but depend on the previous experiences of the agent. Following Gibson's concept of affordances, their schemas do not model a (*context, action*) → *result* relation but rather a (*interraction*_1_, *interraction*_2_) relation. Therefore, the hierarchical structure of schemas' organization is by construction contained in the formalism. The system also uses a representation of the internal state of the agent, the *scope*, which keeps track of the completion of current interactions, hence providing the agent with a simple form of situation awareness.

Guerin and Mckenzie (2008) developed a test bed similar in spirit to Drescher's micro-world (eye-mouth-hand) but based on a 2D physics simulator. Since Piaget's theory is too vague, they argue that computer models should be validated by closely replicating his empirical observations, hence Guerin attempts to replicate the second developmental stage theorized by Piaget. The schemas are inspired by Drescher's schema, but augmented with *target values*: (*context, action*) → (*result, values*), which quantify the value of a schema with respect to subsequently executed schemas. The systems use different types of schemas, including super-schemas, and the learning algorithm presents mechanisms similar to the *options* framework in Reinforcement Learning (Sutton et al., 1999).

The study by Aguilar and Pérez (2017) also seeks to replicate empirical observations on child development, namely the emergence of hand-eye coordination. Their system differs from others in several aspects. The *context*, in addition to sensory features, also represents the emotions and motivations of the agent. The schema learning mechanism is not purely inductive but proceeds through a generalization-splitting-specialization cycle, from which the process of equilibration naturally emerges. The experiments were conducted in a 3D simulated environment more realistic than the aforementioned micro-worlds, but fundamentally, the interaction of the agent with the world happens through discrete steps, using discretized actions and perceptions.

The “Jean System” (Chang et al., 2006) uses the Image Schema Language (ISL) (Amant et al., 2006) to model the knowledge represented by schemas. ISL allows representation of qualitative and dynamic aspects of the relationship between objects, e.g., *A* is *quickly approaching* *B* which is *contained* in *C*. Learning occurs through Experimental State Splitting (ESS), a mechanism that splits states based on minimizing the entropy of the distribution of state transitions, hence maximizing their prediction power. In a subsequent version of the Jean System (Cohen et al., 2007), schemas are built from *controllers* and *maps*. The controllers control actuators, and the maps record sensory traces during the execution of the corresponding schema. In this version of the system, the state splitting mechanism delimits *decision regions* in the maps, from which the system may switch from one controller to another. This differs from previous study in that the system learns its own abstractions from sensory data and operates in the continuous domain.

## 3. Critique of Previous Study

In this section, we elaborate on some areas of divergence between the studies presented above and the approach proposed in this article. The provided references shall help us to define the architectural constraints used to guide the design of the system presented in Section 4. These architectural constraints are of two types: constraints on processes (how the system operates in time) and constraints on representations (how data is represented).

### 3.1. Serial Decision Mechanisms

Our first criticism concerns the way these systems operate in time. In Drescher and Chaput's systems and Georgeon et al.'s systems, the interaction between the agent and the environment is serial and discrete, i.e., it runs through a loop: Perception—Schema selection—Schema execution. This may be due to the limitations of the simple environments they used. The other systems run in more realistic environments, with continuous sensory data and continuous actions. However, their control process remains serial: an action is executed after the completion of the decision process, which occurs after the completion of the perception step. These steps cannot be interrupted nor revised as they occur (Chang et al., 2006 refer to their schemas as finite state machines). In our view, such a control process is not in line with a sensorimotor-based approach. We refer to the Dynamical System approach (Thelen and Smith, 1994) and Embodied Cognition (Varela et al., 1992), which highlight the role of continuous coupling between the agent and the environment. Furthermore, Neuroscience provides numerous accounts of concurrent and continuous decision and planning mechanisms (Cisek and Kalaska, 2010), for simple tasks (Coles et al., 1985; Lepora and Pezzulo, 2015), or more complex behaviors such as hand-writing (Perdikis et al., 2011). It is admitted that low-level cognition is supported by distributed mechanisms, but higher-level tasks are generally thought to require strict serial processing. However, behavioral experiments suggest that some high-level tasks partly rely on concurrent processes (Millroth, 2021).

### 3.2. Global State Representation

The second concern is that all the presented studies make the assumption of a unified representation of the world state. The different sensory modalities are bundled into a single data structure, upon which operations are carried out. This results in several issues: A scaling issue since the dimension of the state space grows exponentially with the number of sensors. Computationally, it makes learning more difficult, unless Machine Learning is used for labeling this large space into a smaller set of relevant contexts. But this leads, in turn, to the *bootstrapping problem* (Drescher, 1991; Kuipers et al., 2006; Mazac et al., 2014), i.e., the fact that learning, if a context is relevant, requires to have labeled it in the first place. Through this example, we point out how the choice of the representation impacts the functional architecture of the system, by imposing specific mechanisms to deal with the problems arising from the chosen representation. Consequently, neither the representation nor the algorithms are biologically plausible. Indeed, it is established that humans and higher mammals use separate visual pathways for perception and action (the “Where” and the “What”; Goodale and Milner, 1992). Besides, a unified representation of the world state is not compatible with some aspects of cognitive development. For instance, it overlooks the process of Sensory Integration, which is a cornerstone of Cognitive and Developmental Neuroscience. Sensory integration is not innate: in both animals and humans, sensory cues are separate or poorly integrated into newborns, and develop through time along with other cognitive skills (Nardini et al., 2010; Lewkowicz and Bremner, 2019).

## 4. Methods

This section describes the minimal system that shall serve as a base for future study. At this stage of our preliminary study, we have not committed to any specific developmental mechanisms. First, we need to observe the consequences of applying biologically plausible constraints on the functional architecture, and then, developmental mechanisms will be devised accordingly. Sensorimotor learning is done offline, so as to provide the agent with a minimal behavioral repertoire. At this stage, our focus is not on the learning process but on answering the question:

Given a repertoire of ~100 trained sensorimotor schemas, which control processes or decisions mechanisms, can be implemented for the robot to act rationally (given a value system) while complying with the following biologically plausible architectural constraints derived from the previous section:

1. the decision process is distributed (Section 3.1).
2. the action currently executed can be refined or changed at any time (Section 3.1).
3. the different sensory modalities are processed separately (Section 3.2).

### 4.1. Simulated Robot and Environment

The environment used for training and testing the system is implemented with the open source 2D physics engine for games Box2d (Catto, 2007). It simulates a top-perspective flat world in which the robot moves under linear and angular damping constraints, which roughly emulates moving through a viscous liquid, i.e., the robot will eventually stop moving if no force is applied to it. The choice of a 2D environment is a compromise between two requirements: the need for a realistic simulation of physics to provide consistent input to the sensorimotor schemas, and the need for a computationally light simulator so that learning can be done efficiently by speeding up the pace of simulation up to 10 times.

The environment is limitless, although there is a central region populated with objects : walls, predators, and prey. Walls prevent the robot's movement, but they do not delimit a closed region, hence the robot is free to move outside the region delimited by the walls. Predators' and preys' motions are ruled by simple controllers. They randomly alternate between straight motions in a random direction or straight motions toward the robot (for the predators) or the central region (for the prey). In this way, although it can reach any location in space, the robot is constantly chased by predators, while it needs to maneuver among the walls where the preys tend to be.

### 4.2. Perception

**Figure 2:**
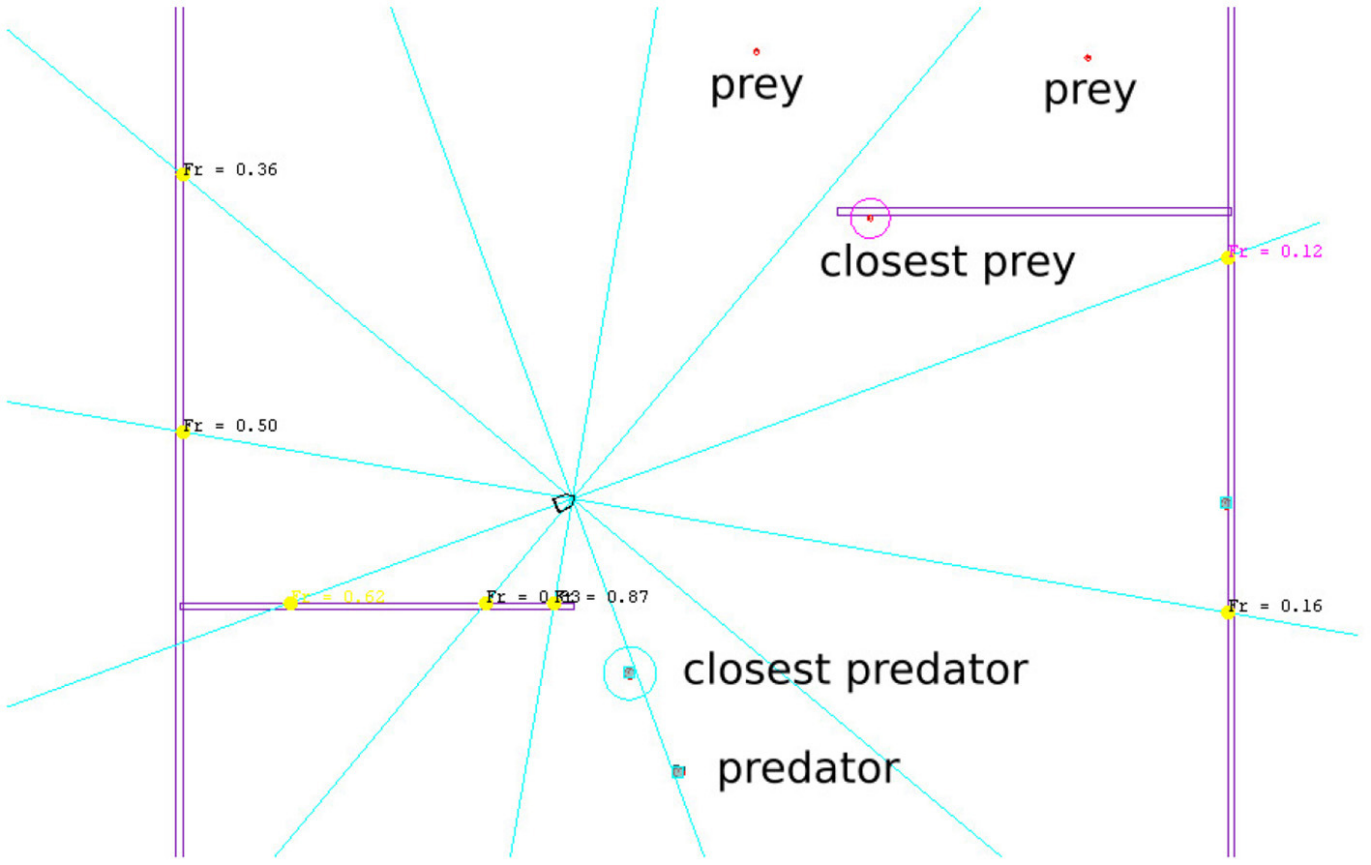
Annotated view of a part of the central region of the simulated environment.

Note: 10% of the sensory data is tampered with using white noise with a magnitude of 10% of the maximal sensory value.

The perception module pre-processes raw sensory data into a lower dimensional input for the sensorimotor schemas. Sensory pre-processing is not done for simplifying the learning problem, but for the sake of realism: natural organisms do pre-process sensory input through specialized structures. The simulated robot perceives its surroundings at 60 Hz through three modalities: distances, prey, and predators:

1. *Obstacle distances:* (*d*_*N*_, *d*_*E*_, *d*_*S*_, *d*_*W*_)Distances to surrounding obstacles are measured by casting 12 rays (one ray every 30°, refer to Figure 2). This raw data is processed by taking the minimum value of three consecutive rays, resulting in four values for North, East, South, and West directions in the frame of reference of the robot.
2. *Closest prey position and velocity:* (*x, y, v*_*x*_, *v*_*y*_)where (*x, y*) is the position vector of the closest prey, and (*v*_*x*_, *v*_*y*_) the corresponding velocity vector, both in the frame of reference of the robot.
3. *Closest predator position and velocity:* (*x, y, v*_*x*_, *v*_*y*_)where (*x, y*) is the position vector of the closest predator, and (*v*_*x*_, *v*_*y*_) the corresponding velocity vector, both in the frame of reference of the robot.

### 4.3. Motor Control

Each schema has its own motor control module, designed to control the motion of the robot with few parameters. To achieve this, actuation has been reduced to a minimum: the robot is a single rigid body subject to a force and a torque applied to its center of mass. The intensity of these forces is controlled by a Dynamic Motion Primitive (DMP) (Ijspeert et al., 2013). A DMP is a dynamical system inspired by the damped spring model:


τý=α(β(g-y)-ẏ)+f,

where α and β are positive constants, τ is the time constant of the system, and *g* is the goal value toward which the system tends to converge. DMPs provide a flexible framework for motion control with well-understood convergence properties and the possibility to be learned through the forcing term *f* (Ijspeert et al., 2013). In the present study, the aim is to produce a reasonable variety of motions using the minimum number of parameters, hence some parameters were held constant (τ = 2, *g* = 0, *f* = 0, α = 8, β = 2), The ratio β = α/4 ensures critical damping of the system, i.e., *y* converges toward *g* without oscillations.

The initial velocity ẏ_0_ was set to 0, hence the system is solely determined by its initial conditions *y*_0_ and smoothly converges toward 0 within a few seconds. The value of *y* is used to control the force and the torque applied to the body of the robot with a frequency of 60 Hz. The actions of the robot are, thus, parameterized by *y*_0_, which will be denoted by *p* in the reminder of the article for convenience. Accordingly, the first component of *p* determines how far the robot moves, while the second component determines how much and in which direction the robot turns.

### 4.4. Sensorimotor Schema

**Figure 3:**
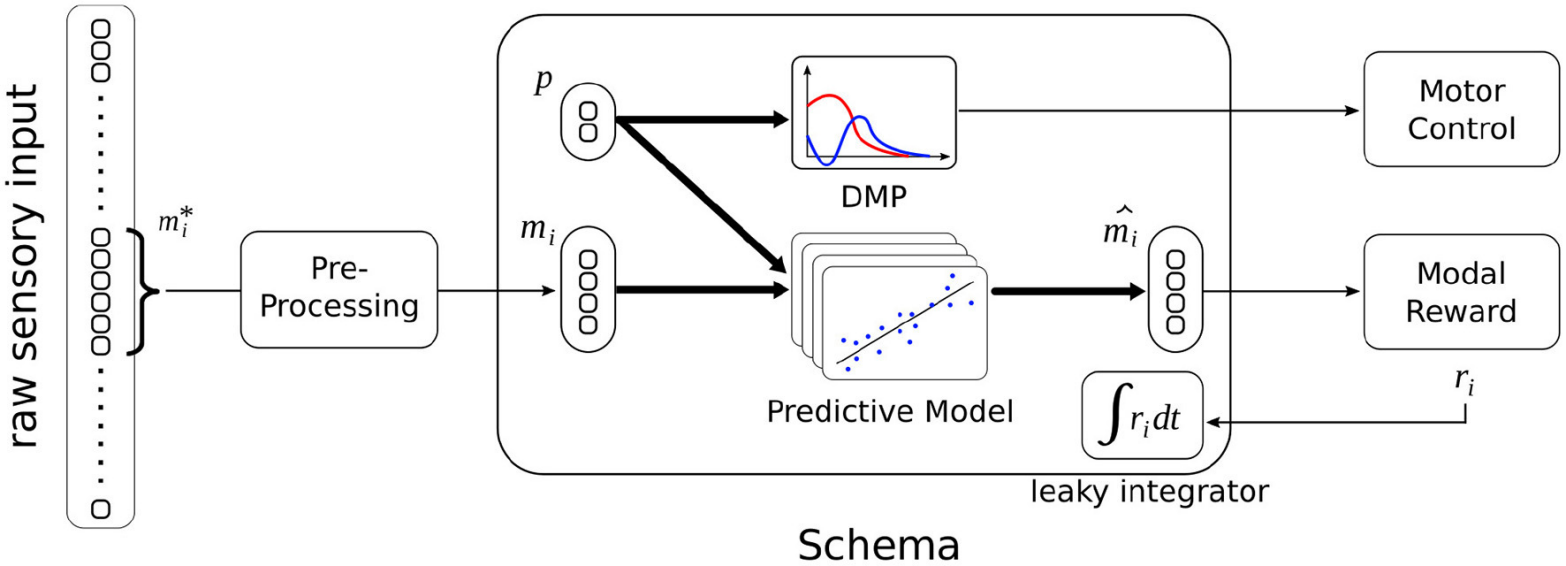
Schematic illustration of the sensorimotor schema in context. Raw sensory data from a specific modality $m_{i}^{*}$ is pre-processed into a lower-dimensional representation *m*_*i*_. After training, given the current modal percept *m*_*i*_ and some action parameterized by *p*, the schema can predict the resulting sensory percept ${\hat{m}}_{i}$, and get the associated reward *r*_*i*_ through the *Modal Reward* component. Some experiments use the leaky integrated value of the reward (refer to Section 4.6). When the schema is triggered, its DMP is used to control the execution of the action.

The proposed schema illustrated in Figure 3 differs from previous study in several aspects. Not only does it operates in the continuous domain, but its *control process* is continuous (refer to Section 4.6), i.e., it can be triggered or interrupted by another schema at any time (within the limit of 60 Hz).

The second difference is that the predictive model does not apply to a full state description but to a single modality. The predictive model consists of multiple linear regressions, where the feature vector is the joint vector (*m*_*i*_, *p*). Each regression predicts a component of the modality ${\hat{m}}_{i}$. The model predicts the value of the modality at the end of the action, i.e., when the DMP has converged to zero. The predictive model does not predict a reward value: there is a *Modal Reward* component that is external, shared by all schemas, and not subject to learning, i.e., it represents an innate system that returns a positive reward for proximity with prey, and negative rewards for proximity with predators and contact with walls.

Another difference compared to previous studies is that the sensorimotor schema here is not tied to a particular *context*, because the notion of context is less relevant when the state is only seen through one modality. Hence, a sensorimotor schema is trained for making predictions over the whole range of possible sensory inputs (within one modality).

### 4.5. Learning

This preliminary study focuses on the sensorimotor layer, hence learning boils down to learning a predictive model for each schema. In the experiments presented in Section 5, learning was done offline for the sake of simplicity, and for avoiding any bias caused by the exploration/exploitation strategy used. The learning method was chosen with the idea of schemas as multiple simple parallel processes in mind. Consequently, a simple (linear) model was chosen, and learning simply consists of a regression of the predicted next sensory state with respect to the current state *m*_*i*_, and some action parameters *p* (refer to below). The main difference with previous study is that the state is not a unique vector encompassing all the modalities: the predictive model only predicts the next state through one modality.

The domain of the feature vector (*m*_*i*_, *p*) was partitioned such that each model learn over a narrow domain, hence representing a particular type of action. The domain was split along *p* only since the notion of “context” is not relevant in the case of a unimodal input. Concretely, an action is parameterized by 2 values *p*_1_, *p*_2_, where *p*_1_∈[0, 1] and *p*_2_∈[−1, 1]. It was empirically found that 0.2 × 0.2 regions lead to good predictions with few training data (<100). Hence, 50 regions were necessary to cover the domain of *p*, thus, 150 models were trained in total (one for each modality).

Learning takes place in the environment as described in Section 4.1, i.e., with predators and prey interacting with the robot. The position of the robot was periodically randomly reset to avoid situations where the robot is “cornered” by predators, hence not providing any novel training data. Training data is collected in a temporary table for the regression process, with a constraint that ensures that no two data are too similar. The training process repeats the following steps until each schema has collected enough data:

1. the sensory state is recorded.
2. an action *p* is randomly chosen and executed.
3. the sensory state is recorded after the DMP has converged.

Since the sensory state contains the three modalities, each executed action provides data for training three models. In fact, the training time is not affected by the number of modalities in this approach, however, it raises a problem of interference between modalities which we discuss in Section 7.

### 4.6. Control Process and Action Selection

Different control processes have been tested, which are all built on a mechanism inspired by the *Affordance Competition Hypothesis* by Cisek (2007). The original model—which applies to neural populations—was adapted to the schema framework. According to this hypothesis, biological brains have evolved to make quick decisions in complex environments presenting multiple choices. The view of serial processing through separate perceptual, cognitive, and motor systems is not supported by recent findings. Instead, phylogeny and neural data suggest that action *selection* and action *specification* occur in parallel, through a competitive process between possible actions, biased by contextual cues.

**Figure 4:**
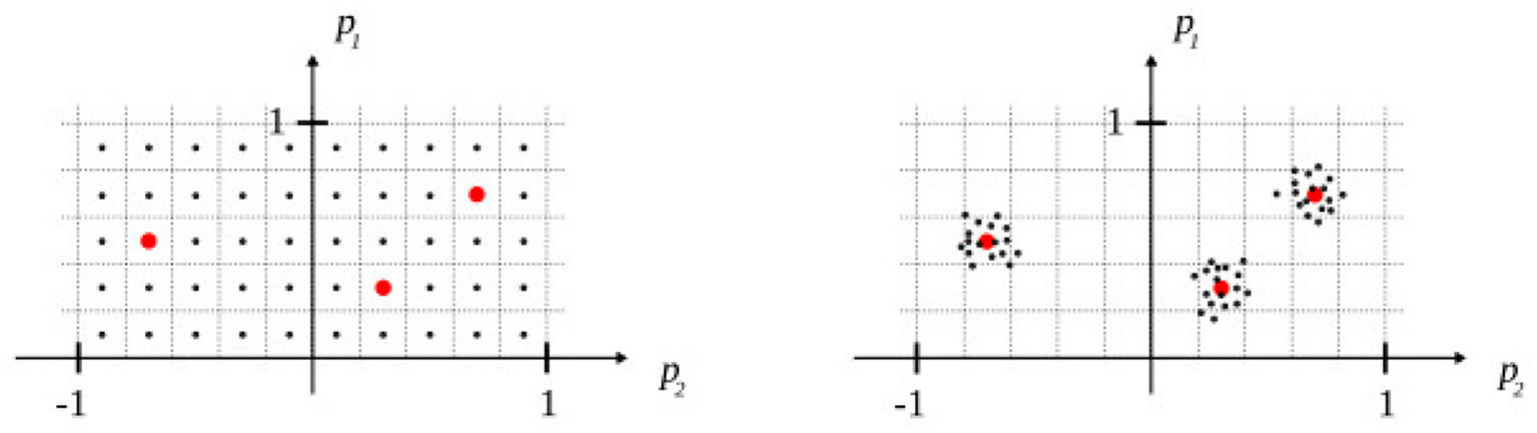
Illustration of the competitive mechanism: global search during the first pass (left), candidates (red) are selected according to a threshold value, and local random search around the candidates during the second pass (right).

This competitive mechanism was functionally emulated with a two-passes search on the space of action parameters (refer to Figure 4). During the first pass, a quick global search is performed to identify the candidate actions (action selection), and during the second pass, a local search is performed to refine the action parameters of candidate actions (action specification). The criteria used by the selection process is ∑*r*_*i*_, the sum of predicted rewards in each modality, i.e., for a given state *s* and an action parameter *p*, three schemas are used: one in each modality, whose region contains *p*. Then each schema makes a prediction in its respective modality and the Modal Reward system computes a reward value for each of them:


$$\begin{array}{l} sh_{1}:\left(m_{1},p\right)\to {\hat{m}}_{1}\to r_{1}\\ sh_{2}:\left(m_{2},p\right)\to {\hat{m}}_{2}\to r_{2}\\ sh_{3}:\left(m_{3},p\right)\to {\hat{m}}_{3}\to r_{3} \end{array}$$

Another feature of Cisek's model and similar decision models is the accumulation of information through time (Usher and Mcclelland, 2001; Mazurek et al., 2003). To emulate this, each schema is endowed with a leaky integrator fed by the reward value predicted at each time step. The decision of triggering the execution of a schema is made when the sum of the integrators' values in each modality reaches a given threshold.

## 5. Preliminary Experiments

The presented experiments are not meant to support the proposed constraint-based method with experimental data, but rather to gain insight into what these constraints imply for the practical act of designing algorithms, which shall guide us in the future development of the system.

### 5.1. Experimental Setup

Different control processes have been compared, which differ in the way they unfold in time or in the way actions are selected. They comply with the constraints listed in Section 4, except the *serial* control process which does not comply with constraint #2:

- **Serial:** A new schema is selected and executed *after* completion of the currently executing schema (similar to the control processes used in the reviewed literature).
- **Continuous:** A new schema can be selected and executed *while* the current schema is being executed.
- **Continuous with leaky integrator:** Same as the “continuous” scheme, but using the leaky-integrated value instead of the instantaneous value of the predicted reward.

Each control process was evaluated by letting the agent act freely in the simulated environment for 10 min, during which the robot could execute 250 actions on average. The performance was measured by the accumulated reward during this time.

For informal comparison, we implemented a decision mechanism based on a traditional AI planning technique: forward search in the space of states. This approach does not comply either with constraint #3, since the basic operations of the algorithm apply to a global state representation:

- **Serial with the forward search:** Same as the “serial” scheme, but performs a random forward search. The predicted state is used as a starting state to make 20 predictions of the next state using random action parameters and select the action leading to the best resulting state. A version looking three steps ahead was also tested.

## 6. Results

**Figure 5:**
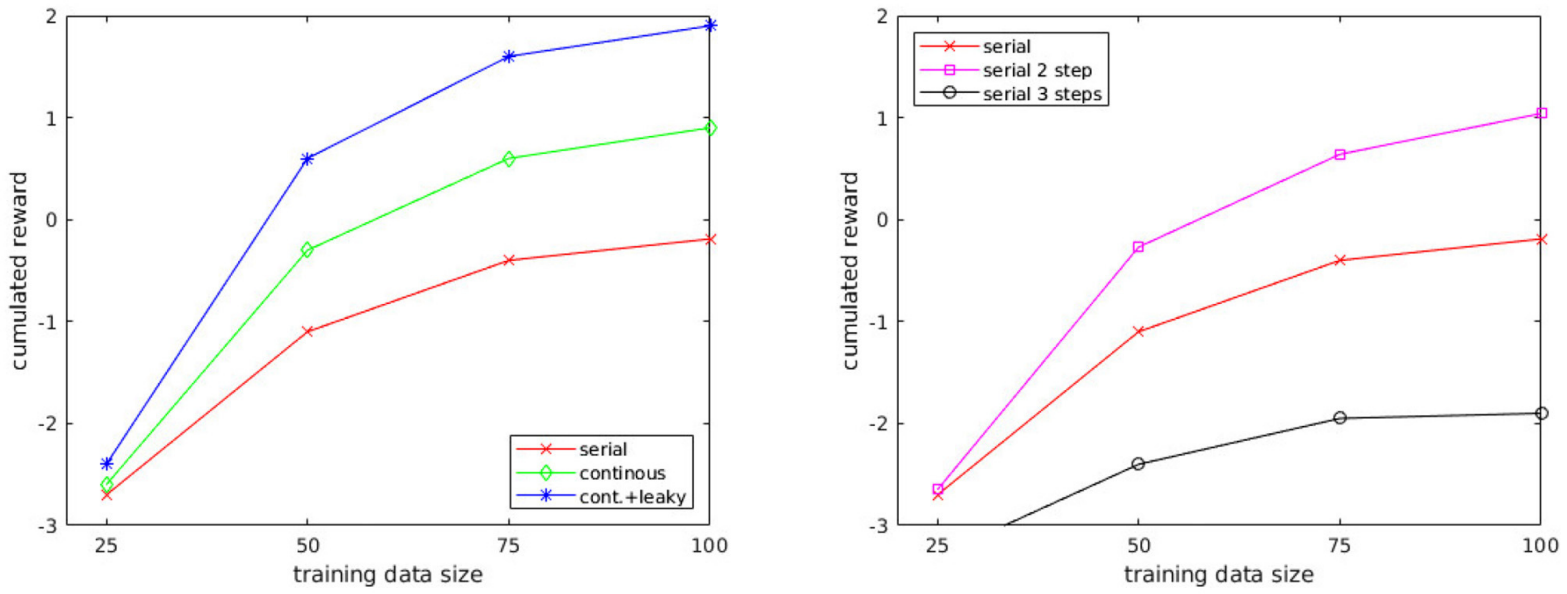
(Left) Comparison of different control processes: serial, continuous, continuous with leaky integrator. (Right) Comparison of the serial control process with different depths: predicting 1, 2, and 3 steps ahead.

The results of comparison between different control processes (Figure 5, Left) were expected. The continuous control process is superior to the discrete one, which does not have the capacity to revise its decision as the action is being executed. Hence, if the action was taken on the wrong premises (e.g., noise, predator not perceived), the agent needs to wait for the completion of the action to change or correct its action. However, the continuous control process is not completely immune to noisy input, and it also causes the agent to frequently “change its mind,” e.g., it can move toward prey and miss the reward at the last moment because of reacting to an approaching predator. The control process using the leaky integrator is more consistent in its decisions since changing behavior requires the accumulation of contradictory input over a certain amount of time. Supposedly, it also has a smoothing effect on noisy sensory data. The only drawback is that the time constant of the integrator has to be manually set, but the adjustment of the value needs not to be precise (any value between 0.25 and 1*s* would give similar results in our setup).

Another result from these experiments concerns the amount of training data. Few data are needed (above 100 samples, little improvement was observed). This was expected considering the low dimensionality of the data due to the multi-modal representation. It should be noted that even with 25 training samples, the cumulated reward is higher compared to the cumulated reward using random actions (which is −5).

The comparison of serial decision mechanisms using different depths (Figure 5, Right) shows that looking 2 steps ahead improves the performance of the agent. Indeed, only predicting the next state makes the agent blind to the states which are not reachable by its action repertoire. As the motion primitives represent short actions, their duration ranges from 0.5 to ~4*s*, hence, the agent can only move up to ~6 m in the course of one action. Thus, if the closest prey is located beyond this distance, no schema can predict a state in which the robot is nearby the prey, i.e., a rewarding state. However, it becomes possible by looking 2 steps ahead, which explains the improvement, but the drawback of this technique is that prediction errors accumulate very quickly. It is still advantageous at two steps, but looking three steps ahead results in completely distorted predictions, and the advantage of a longer planning horizon is lost. On a side note, looking three steps ahead comes with a 20-fold increased computational cost compared with two steps, which did not allow us to run the simulation in real-time.

## 7. Discussion and Future Study

The preliminary experiments presented above were a practical application of the method proposed in the motivation of this article, i.e., complying with constraints imposed by the functional architecture of biological brains. These experiments do not validate the method, but they are a proof of concept for a simple cognitive agent which does not resort to the tenets of classical methods: global state representation and discrete serial processes. Therefore, we hope to encourage further research on schema-based approaches with a scientific method inspired by model-based cognitive science which stands on stronger foundations than the functional modeling approach.

The next step is to devise some developmental mechanisms that comply with these constraints. Note that these constraints are not cast in stone: they might be updated as the functional architecture of the brain is better understood. Before addressing developmental mechanisms, we have to reflect on the fact that some issues may not have come forward because of the simplicity of the system. For instance, the fact that the motor system only controls one effector eludes the problem of interference between concurrent actions. Therefore, augmenting the system with an additional effector, but also enriching sensory input with additional modalities, should be the very next step. This will certainly entail the need for executive functions such as selective attention (Cohen and Magen, 2004) or inhibitory control, although inhibitory control seems to be a skill that develops through time (Dempster, 1992; van der Molen, 2000).

Two approaches for developing higher-order functions from primary functions are commonly proposed: abstraction and concatenation (e.g., Drescher's, 1987 synthetic items and composite actions). We shall follow a similar line of thought, but we anticipate implementing radically different mechanisms because our system processes sensory modalities separately at the sensorimotor level. This constraint naturally leads us toward a developmental mechanism that accounts for sensory integration, which is consistent with the fact that multisensory processes are elaborated through development (Lewkowicz, 1992; Lewkowicz and Bremner, 2019). This last point shows an expected feature of our method: by constraining us to implement the system in a specific way, any new mechanism integrated into the system is *by design* likely to be consistent with biological data. This is encouraging for future study.

There are other directions to explore: we might consider (Vernon et al., 2016) desirata for developmental cognitive architectures in our framework because it complements our approach with top-down constraints. The architectural constraints presented in this article constrain the types of mechanisms that can be implemented from the bottom up, while Vernon's desirata provide top-down constraints, i.e., a set of *necessary* functions for biologically-inspired developmental systems. We shall also examine the study by Karmiloff-Smith, who introduces the Representational Redescription conceptual mechanism (Karmiloff-Smith, 1996), a biologically plausible developmental mechanism from the perspective of representations.

Another major pitfall to avoid is to exclusively look at cognition through the lens of development, and overlook the role of innate priors (Leslie, 1982; Ullman et al., 2012), which might make the problem more challenging than it is. As a result, we will follow the proposed method: starting with a simple system, and incrementally adding functions through mechanisms that comply with biologically plausible constraints.

## Data Availability Statement

The original contributions presented in the study are included in the article/supplementary material, further inquiries can be directed to the corresponding author/s.

## Author Contributions

The author confirms being the sole contributor of this work and has approved it for publication.

## Funding

This study was supported by the Swedish Knowledge Foundation (KKS) synergy project TeamRob.

## Conflict of Interest

The author declares that the research was conducted in the absence of any commercial or financial relationships that could be construed as a potential conflict of interest.

## Publisher's Note

All claims expressed in this article are solely those of the authors and do not necessarily represent those of their affiliated organizations, or those of the publisher, the editors and the reviewers. Any product that may be evaluated in this article, or claim that may be made by its manufacturer, is not guaranteed or endorsed by the publisher.
